# Anchor Loss Reduction in Micro-Electro Mechanical Systems Flexural Beam Resonators Using Trench Hole Array Reflectors

**DOI:** 10.3390/mi14112036

**Published:** 2023-10-31

**Authors:** Mohammad Kazemi, Seyedfakhreddin Nabavi, Mathieu Gratuze, Frederic Nabki

**Affiliations:** Department of Electrical Engineering, École de Technologie Supérieure, Université du Québec, Montréal, QC H3C 1K3, Canada; seyedfakhreddin.nabavi@lacime.etsmtl.ca (S.N.); mathieu.gratuze.1@ens.etsmtl.ca (M.G.); frederic.nabki@etsmtl.ca (F.N.)

**Keywords:** beam resonators, acoustic wave reflector, quality factor, anchor losses

## Abstract

The quality factor of microelectromechanical resonators is a crucial performance metric and has thus been the subject of numerous studies aimed at maximizing its value by minimizing the anchor loss. This work presents a study on the effect of elastic wave reflectors on the quality factor of MEMS clamped–clamped flexural beam resonators. The elastic wave reflectors are a series of holes created by trenches in the silicon substrate of the resonators. In this regard, four different shapes of arrayed holes are considered, i.e., two sizes of squares and two half circles with different directions are positioned in proximity to the anchors. The impact of these shapes on the quality factor is examined through both numerical simulations and experimental analysis. A 2D in-plane wave propagation model with a low-reflecting fixed boundary condition was used in the numerical simulation to predict the behavior, and the MEMS resonator prototypes were fabricated using a commercially available micro-fabrication process to validate the findings. Notably, the research identifies that half-circle-shaped holes with their curved sides facing the anchors yield the most promising results. With these reflectors, the quality factor of the resonator is increased by a factor of 1.70× in air or 1.72× in vacuum.

## 1. Introduction

MEMS resonators have been used for a wide range of applications such as gas sensing [1,2], frequency tracking [3], magnetic sensing [4], and thermo-humidity sensing [5]. The quality factor (*Q*) of a resonator is a crucial metric that quantifies the overall performance of the device. (*Q*) is a dimensionless parameter that describes the energy stored in the resonator divided by the energy lost per cycle, and can be expressed as [6]:

(1) $$Q=2\pi \frac{W}{\Delta W},$$

where *W* and Δ*W* denote the maximum stored energy in the resonator structure and the dissipated energy during each vibration cycle, respectively. There are different mechanisms that lead to the dissipation of energy in a resonating system. The total *Q* of a resonator can be determined by the reciprocal summation of the *Q* of all of the energy loss mechanisms [7] such that:

(2) $$\frac{1}{Q_{total}}=\frac{1}{Q_{air}}+\frac{1}{Q_{TED}}+\frac{1}{Q_{surface}}+\frac{1}{Q_{anchor}}+\frac{1}{Q_{others}}.$$

Each term of (2) relates to an energy loss mechanism. $Q_{air}$ denotes the air or viscous energy loss which occurs due to the compressive force of the peripheral medium (e.g., air) onto the surface of the resonator [8,9], $Q_{TED}$ is the thermo-elastic damping that is caused by the induced temperature gradient by the mechanical vibration of the resonator [10,11,12], and $Q_{surface}$ is the surface loss that is caused by the existence of a thin layer of contaminations and defects on the surface of the resonator [13]. In addition, Akhiezer damping [14] is another source of energy loss in resonators comprehensively studied by [15]. Finally, $Q_{anchor}$ is due to the energy loss through the anchoring structures into the substrate, which is one of the main sources of energy loss in resonators [16].

In vacuum, where the air damping is diminished, the main energy losses occur through the anchors and thermo-elastic damping [17]. The analytical modeling of the anchor loss has been extensively employed in order to minimize energy dissipation. Qualitative analytical and quantitative numerical methods were proposed by [18] to predict the anchor loss of AlN 65 MHz contour mode resonators considering the anchor as a 1D waveguide. A theoretical model for the anchor loss of flexural beam resonators with in-plane vibration was presented by [19]. The effect of different design parameters such as different electrode patterns, as well as the curvature of the resonating structure’s edges on the *Q* of AlN on silicon Lamé mode resonators, were studied using the perfectly matched layer (PML) method by [20]. Moreover, the effect of anchor geometry on the *Q* of width extensional mode resonators at 52 MHz were modeled in [21] using a PML. This work confirmed that the anchor geometry has a significant effect on the *Q* of these types of resonators. Notably, many other works have widely discussed analytical and numerical models in order to predict the anchor loss and *Q* of different types of resonators [22,23,24,25,26,27].

In addition to studies on modeling the anchor loss, many studies have investigated different methods to increase the *Q* of resonators by decreasing the anchor loss. A well-known method for increasing the *Q* is using wave reflectors [28] which can be created by trenching the substrate layer to reduce the wave dissipation per cycle into the substrate. It is shown that acoustic in-plane reflectors were employed to increase the *Q* of lateral extensional MEMS resonators [29]. Phononic crystals (PnC) are materials that have their acoustic properties periodically changed in a certain structure and either hinder or allow the propagation of waves in a certain frequency range. This feature of PnCs makes them useful for reducing the anchor loss in resonators and thus, many researchers have used different 1D and 2D shaped PnCs for the anchor loss reduction [30,31,32,33,34,35]. More recently, [36] proposed different types of phononic crystals on the anchor structure, which resulted in an increase in the *Q* by up to 230%. In another study, [37] increased the *Q* of a dome-shaped resonator by isolating the structure using trenched areas around the resonator.

It should be mentioned that, despite the fact that PnCs demonstrated their effectiveness at mitigating anchor loss, they require a relatively complex structural design based on an acoustic bandgap. Thus, the effectiveness of PnC-based devices is highly dependent on the precision of the fabrication process.

Generally, acoustic reflectors are designed to obstruct the propagation of energy, whereas the comprehensive evaluation of these reflectors in terms of their energy-related functionalities has remained unexplored in the literature. Furthermore, a substantial part of the literature that is related to anchor loss reduction and its associated design has predominantly centered around the modeling of PnCs. As a result, alternative methodologies that offer unique advantages such as simplicity have received relatively less attention. This emphasizes the necessity for further research to investigate diverse strategies for predicting and mitigating anchor loss, aligning with the primary objective of this study. Therefore, this work aims to investigate the effect of different shape trench hole arrays on the energy loss and *Q* of clamped–clamped beam resonators, and explore the design parameters suited for maximum improvements in performance.

Accordingly, this work presents a study of the effect of the trench hole arrays of various geometries acting as reflectors on the performance of clamped–clamped flexural beam resonators. These reflectors reduce the wave propagation from the anchors of the resonators to the substrate. A 2D model is used to simulate wave propagation in the anchoring structure of the resonators. The anchors are patterned with a series of arrayed holes of different shapes, located near the fixed ends of the resonators. Then, in order to enhance the *Q* of the clamped–clamped flexural beam resonators, the presented model is employed to assess various trench hole array geometries. These holes have an effect on the resonator’s energy loss and can enhance the *Q*, as demonstrated. The PML model is also utilized to conduct an analysis on the *Q* of resonators with reflective holes on anchors with different shapes and sizes. Thereafter, the modeled resonators are fabricated utilizing the PiezoMUMPs bulk micro-machining technology from MEMSCAP in order to experimentally validate the numerical simulations. It is found that certain hole array geometries result in an increase in *Q*, while the resonant frequency is varied minimally.

The design and then the fabrication process of the resonators and the structure of the reflectors are presented in Section 2. The numerical simulations based on 2D wave propagation and a PML model are presented in Section 3. This is followed by the experimental measurement of the fabricated devices in Section 4. Finally, a conclusion is presented.

## 2. Micro-Fabrication Process and Design

### 2.1. Fabrication Using the PiezoMUMPs Process

**Figure 1:**
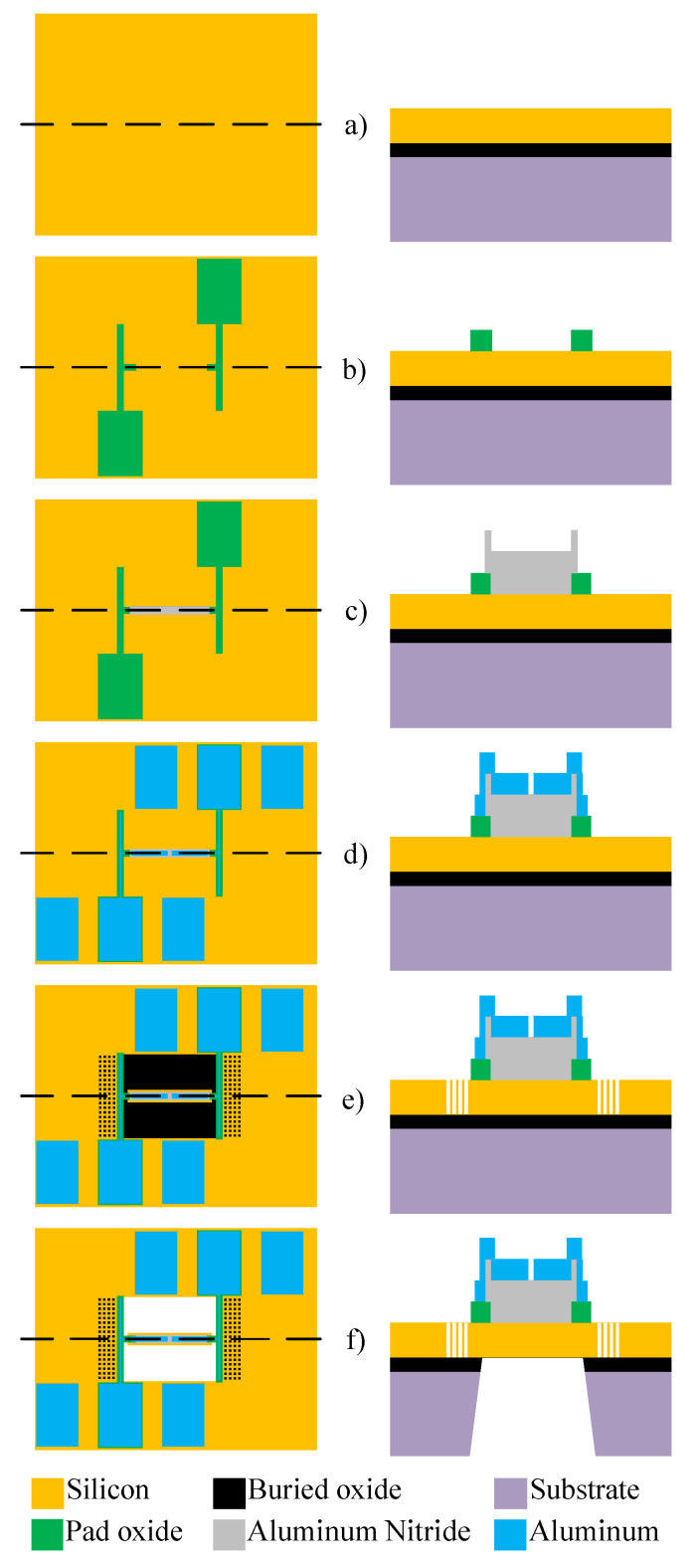
Overview of the PiezoMUMPs fabrication process used to create the clamped–clamped MEMS resonators; (**a**) SOI wafer with 10 μm-thick top-doped device layer, (**b**) patterning and deposition of the silicon dioxide insulating layer; (**c**) AlN piezoelectric film deposition and patterning; (**d**) Al layer deposition and patterning; (**e**) device layer patterning; and (**f**) etching of the trench.

For the experimental validation of the studied resonators, the PiezoMUMPS process from MEMSCAP, Crolles, France, was selected. In this fabrication process flow, as depicted in Figure 1, an N-type silicon on insulator (SOI) wafer which is double-sided polished is used. The wafer has a 400 μm-thick substrate (handle layer) and a 10 μm-thick conductive silicon device layer due to top-side doping. A thin layer of 0.2 μm-thick thermal oxide of SiO${}_{2}$ is deposited and patterned onto the wafer as the first step as an insulating layer. This layer provides the isolation of the device layer from the Al metal layer. The process is followed by the deposition of the 0.5 μm-thick piezoelectric layer which is AlN. This layer is patterned using a wet etch process. In order to form the electrical interconnect, a stack consisting of 20 nm-thick chromium (Cr) and 1 μm-thick aluminum (Al) is patterned using the lift-off process. The 400 μm-thick bottom silicon substrate is then patterned and etched in order to create a trench and release the mechanical resonator so that it can move. Thus, the released resonators can vibrate under the effect of an excitation signal applied to the top interconnect and the device layer that together sandwich the piezoelectric film. More information on this fabrication process is given in [38].

### 2.2. Resonator Design Overview

**Figure 2:**
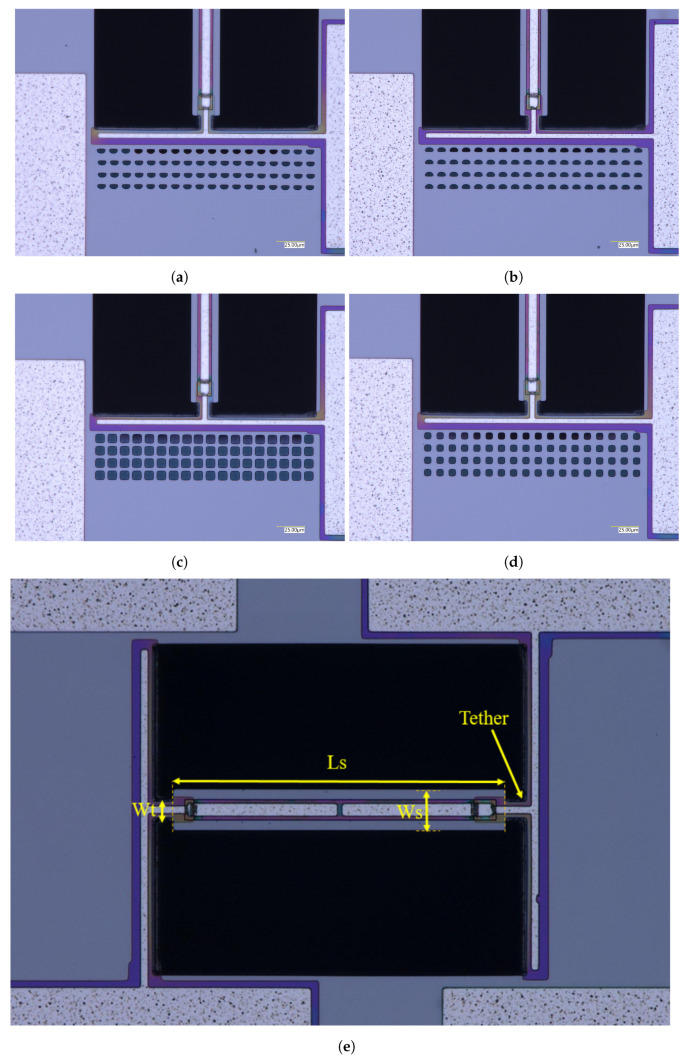
Scanning electron microscope (SEM) micrographs of the fabricated clamped–clamped beam resonators showing different trench holes on the substrate forming the arrayed-holes reflectors. Different hole geometries were fabricated along with a reference device: (**a**) half-circle towards the resonator (named half-circle-line); (**b**) half-circle away from the resonator (named half-circle-curve); (**c**) large square; (**d**) small-square; and (**e**) reference device with no reflector.

The fabricated clamped–clamped beam resonators, as shown in Figure 2, have dimensions of 225 μm in length ($L_{R}$) and 25 μm in width ($W_{R}$). The tether length on each end of the beam is 12.5 μm. Table 1 summarizes the device dimensions. It is worth pointing out that the energy dissipation mechanism due to the anchors in the resonator primarily occurs through the tether, resulting in an inversely proportional relationship between the tether width and the *Q*.

As the width of the tether increases, the surface area exposed to the surrounding medium increases, leading to an increase in the energy loss and a decrease in the *Q*. Conversely, a reduction in tether width results in a decreased surface area and lower energy dissipation, thus resulting in an improvement in the *Q* and increased energy storage capacity. Accordingly, in order to reduce the energy loss due to the anchors, the width of the tether is set to be 10 μm, which is chosen based on the minimum allowed dimension by the design rules of PiezoMUMPS. This minimizes the escape of elastic waves from the resonator and decreases the *Q* reduction. The boundary of the trench below the resonator is set to be at the start of the reflector structure.

To create the reflectors, arrays of identical holes are defined, consisting of 4 rows of 18 columns, with the holes having a center-to-center spacing of 20 μm in both the x and y directions. These arrays are placed on the fixed substrate 20 μm away from the tether. We could observe the benefits of adding more rows of holes in reducing the anchor loss for up to four rows. Beyond four rows, improvements were not seen. Additionally, increasing the hole size reduces the anchoring structure stiffness, lowering the resonator’s resonant frequency significantly, which can be a detrimental effect of the proposed technique. Hence, we limited the hole sizes considered in this study to stay below about 10 μm. To investigate the effect of the reflectors’ shape on the wave reflection and the *Q* of the resonators, several beam resonators were fabricated with different shapes of holes. The following shapes are considered: half-circles with a diameter of 8 μm facing toward the resonator (named half-circle-line), as shown in Figure 2a, similar half-circles facing away from the resonator (named half-circle-curve), shown in Figure 2b, large 8 μm-wide squares (named large square), shown in Figure 2c and smaller 6 μm-wide squares (named small square), shown in Figure 2d. A reference device consisting of a clamped–clamped resonator is also created without holes in order to compare the effects of the hole arrays on the anchor losses, as shown in Figure 2e, along with the key dimensions outlined.

## 3. Simulation Results

### 3.1. Wave Propagation

The vibration of the flexural beam resonators generates elastic energy in the anchors of the resonator. This elastic energy travels to the substrate through the anchors and the dissipation of this energy leads to the energy loss that reduces the *Q* [39].

**Figure 3:**
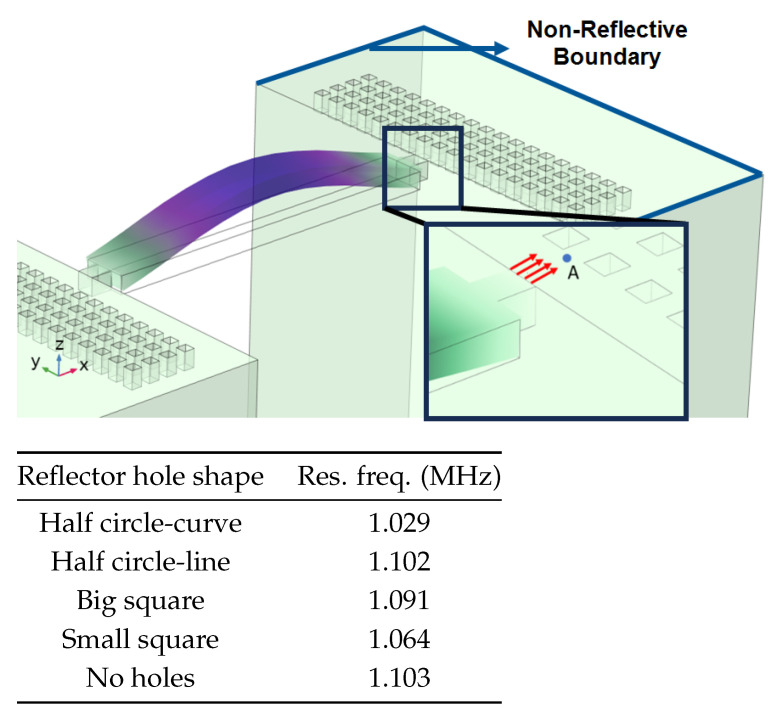
Representation of the mode shape of the resonator, placement of the reflective arrayed holes and the non-reflective boundaries. Red arrows represent the energy loss direction with the table showing the frequency of each design. Simulated resonant frequencies are also indicated for different reflector geometries.

According to beam theory, the displacement and velocity are assumed to be zero at the anchor locations of a clamped–clamped beam resonator. However, the vibration of the resonator generates an alternating stress at this location (i.e., anchor points). This results in an elastic wave that propagates and dissipates into the substrate, leading to anchor-related energy losses in the resonator. This behavior is graphically shown in Figure 3, where the numerically extracted resonant frequencies of the resonator with the reflectors of different geometries on their anchors are also shown.

In this section, the numerical analysis of the energy dissipation was carried-out using the COMSOL Multiphysics software package in the time-domain to investigate the energy dissipation behavior for the various anchoring structures. First, it is assumed that the elastic wave that travels into the substrate in the z direction will not reflect back to the resonator, and will be dissipated into the thick substrate. It is worth mentioning that the substrate is assumed as an infinite medium in the simulation study. Furthermore, the energy that passes the reflective holes is also considered to travel to the infinite medium and be dissipated.

**Figure 4:**
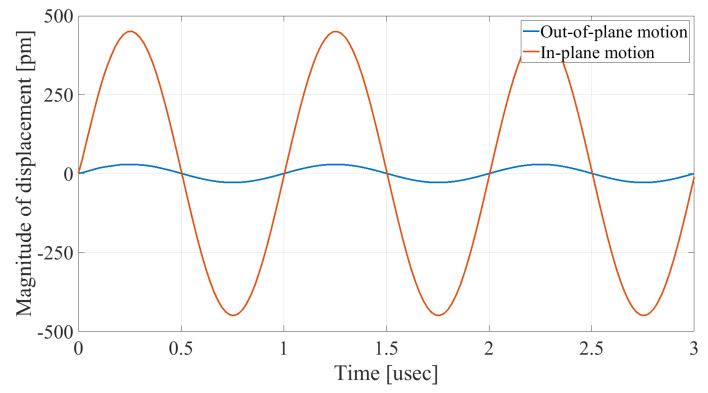
Comparison of the vertical and lateral displacement at the anchor.

In order to demonstrate that the initial postulation mentioned above is valid and that the out-of-plane displacement and velocity are assumed to be zero at the anchors, the beam was electrically excited at its first resonant mode and the deformation of the anchoring point, labeled in Figure 3, was computed along the x axis and z axis. With reference to Figure 4, it can be seen that the out-of-plane displacement (i.e., z axis) of point “A” on the anchor is significantly smaller than the displacement obtained along the lateral axis (i.e., x axis). This is in agreement with our hypothesis that the out-of-plane motion and stress in the anchors are negligible compared to the in-plane motion and stress. Therefore, it is possible to simplify the simulation to a 2D numerical analysis and the anchor can be considered as a thin plate with a thickness of 10 μm. In order to enhance the accuracy of the model, the mesh size is chosen to be 2 μm, using a free triangular mesh. The stress generated by the vibration of the resonator will travel from the anchor points.

**Figure 5:**
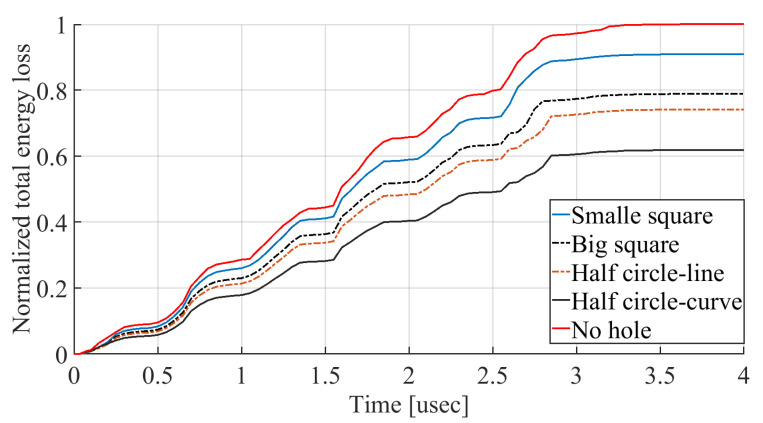
Normalized total energy transferred to the substrate over time for the different reflector geometries studied.

The total amount of energy that passes the reflective holes and reaches the non-reflecting boundaries can then be indicative of the energy loss in a resonator. The energy that passes through the reflectors is considered to be dissipated and lost into the substrate. In order to mimic this dissipation of the energy, a non-reflecting boundary is employed at the vicinity of the anchor, as shown in Figure 3. As such, no wave can be reflected back to the vibrating beam after passing the holes. In this finite element method (FEM) analysis of the wave propagation in the anchors, the amount of elastic energy that passes the substrate and reaches the non-reflecting boundary represents the dissipated energy. This was observed in a 2D medium where the total energy lost in the non-reflecting boundary was quantified when the anchor point was excited by three periods of a sinusoidal force. This quantification is performed using boundary probes at the non-reflecting boundaries and the derivation of the total energy in a dynamic solution The four different reflector geometries were studied with this method. It should be noted that the sinusoidal forces are exerted at the resonant frequency of each structure, as listed in Figure 3. The results of this computation are shown in Figure 5 where, in order to provide a representative comparison, the total energy losses of the resonators are normalized to the resonator with no hole in its anchoring structure.

According to Figure 5, the results indicate that, among the resonators evaluated, the one without a reflector had the highest overall energy loss, while the resonator with holes in the shape of a half-circle-curve has the lowest energy loss. This suggests that the reflective holes impede wave dissipation and that the half-circle-curve shaped holes have the most efficient wave reflection properties.

To further demonstrate the effectiveness of the employed reflective holes for each standalone resonator, the wave propagation in the anchoring structures is graphically illustrated in Table 2. The data clearly demonstrate that the absence of holes on the anchoring structure of the resonator leads to a significant loss of energy as the wave travels towards the substrate without reflection and ultimately dissipates. In contrast, the presence of holes in the anchoring structure of the resonators effectively reflects the wave back towards the energy source, resulting in a lower energy loss.

Furthermore, it can be deduced that the shape of the holes plays an important role in the effectiveness of the arrayed hole reflectors. The half-circle-curve shape, in particular, is observed to be highly effective in reflecting the energy back toward the resonator. This highlights the importance of considering the geometric properties of the reflector holes in the design of resonators to minimize energy loss.

### 3.2. Perfectly Matched Layer

This section serves to present the results of the numerical analysis of the resulting *Q* of the clamped–clamped resonators with the different reflectors. The perfectly matched layer (PML), which is a well-known numerical method to predict the *Q* of resonators, was proposed by [40] for electromagnetic wave problems. The concept of a PML is to create an artificial boundary layer around the simulation domain that absorbs the outgoing waves, mimicking the behavior of an infinite domain. The method was later expanded and re-integrated for wave-like equations [41], and applied to curvilinear coordinates [42]. Afterwards, the PML method was developed for complex space coordinates [43] and later on for anisotropic heterogeneous media [44] that act as an infinite domain, and eliminate the reflections of the lost waves back towards the resonator. Due to the limitations in fully modeling the substrate, researchers often rely on PML as a way to measure the anchor losses in MEMS resonators [21,45]. This method is based on the FEM, with the PML serving as a simulated boundary layer that has the ability to absorb the energy of the acoustic waves coming from the resonating MEMS device [46].

In order to assess the potential impact of reflective holes on the energy loss of the clamped–clamped resonators, we employed this method utilizing the PML option in the COMSOL Multiphysics software package. The method of stretched coordinates is employed in COMSOL to dampen the waves traveling perpendicular to the PML device interface. In order to attain the most accurate results, the meshing for the PML region consists of a swept mesh, incorporating 25 layers. The PML region is stretched using the default coordinate stretching option (i.e., polynomial). The PML scaling factor and curvature parameter are both set to the default value of 1. It should also be mentioned that neither of these analyses incorporated thermoelastic damping or air damping, and our primary focus is on mechanical loss due to anchor loss. Additionally, anchor loss is known in vacuum to be the main source of energy loss, dominating the *Q* performance in such flexural mode resonators.

**Figure 6:**
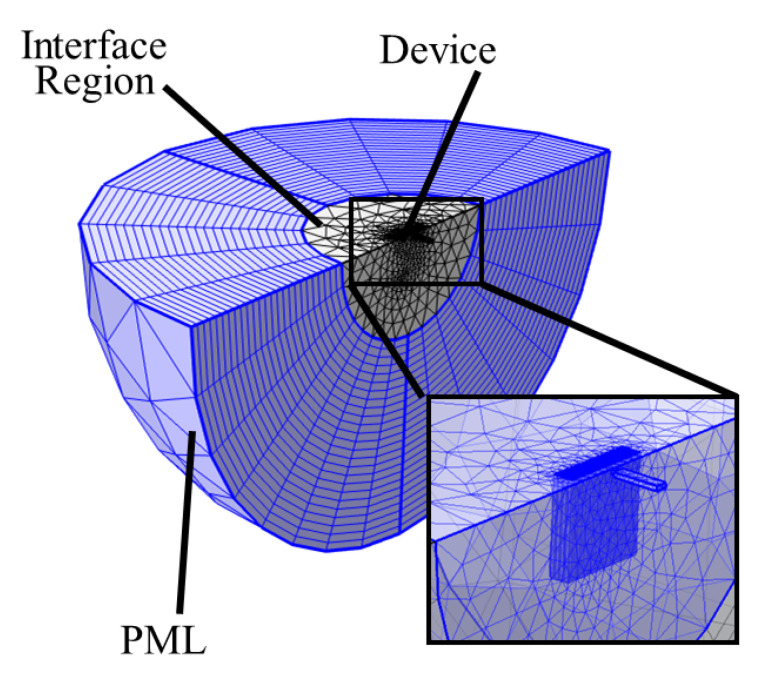
Anchor loss model implemented in COMSOL showing the resonator device, interface region, and the PML area, with the fine mesh used at the resonator periphery shown in the inset.

Figure 6 shows the positioning of the resonator with symmetry on a hemispherical PML. In order to obtain the best matching results, the hemispherical PML diameter (*d*) is set to 10.5 mm as the PML size should be 20% greater than the wavelength in silicon at the resonance frequency, i.e., 1.1 MHz [47]. In addition, the mesh is more finely defined in the vicinity of the resonator, notably on the anchoring structure, as shown in the figure inset. Table 3 presents the results of the PML analysis for different anchor types, showing that the PML predicts that placing holes on the anchors increases the *Q*, with varying effectiveness for each hole geometry. This is consistent with the results of the previously discussed 2D wave propagation model. When observing the *Q* extracted using the PML 3D model, and assuming that the anchor loss is the dominant loss mechanism, it becomes apparent that the simpler 2D model accurately predicts the anchor loss trend between all of the studied hole geometries. In fact, the PML model predicts that the *Q* of the resonator with a half-circle-curve geometry is the highest while the resonator with small-square-shaped holes has the least improvement in its *Q*. As expected, all of the reflectors yield a better *Q* than the structure without holes.

In order to readily compare the *Q* extracted from the PML analysis to the measurement results later in this paper, these results are included in Table 4 as $Q_{PML}$.

## 4. Experimental Results

### 4.1. Description of the Experimental Test Setup

**Figure 7:**
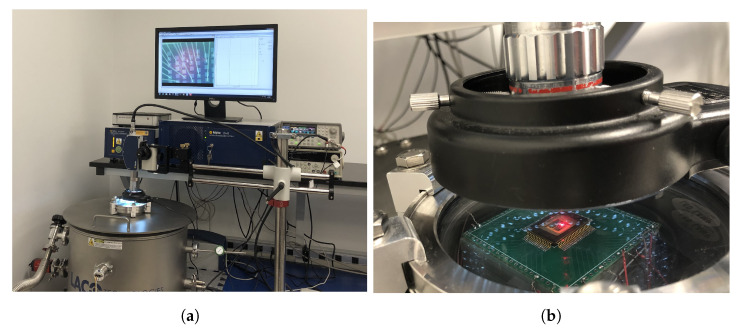
Picture of the vibrometer test bench (**a**) wide view; and (**b**) zoomed-in view of the device in the vacuum chamber.

In order to compare the results obtained from the 2D wave propagation and PML models for anchor loss, frequency response measurements were conducted on the fabricated resonators. In this study, a vibrometer (OFV-2570 Polytec controller and the Polytec laser OFV-534) were used to measure the frequency responses of the prototyped resonators. From the measured frequency responses, the mechanical *Q* of the resonators were calculated. Figure 7 depicts the test setup for measuring the frequency and *Q* of the studied MEMS resonators. The device is placed inside the vacuum chamber, which minimizes the effects of air damping on the measurements. The signal generator is connected to the device and provides the excitation signal for the resonator. The vacuum chamber is used in order to create an environment with minimal air damping to obtain accurate measurements of the frequency and *Q* of the resonator.

The vibrometer is able to characterize out-of-plane motion. The excitation of the device beam resonators is performed using a 33,250 A Keysight function generator that is used to sweep a sinusoidal excitation frequency from 0.9 MHz to 1.2 MHz with an amplitude of 20 V peak-to-peak. The vibrometer laser is focused on the midpoint of the resonators where the maximum displacement occurs in the first mode shape of the clamped–clamped resonators. In order to eliminate the presence of air damping in the measurements, the pressure of the chamber was reduced to 3 mTorr.

### 4.2. Anchor Stiffness

**Figure 8:**
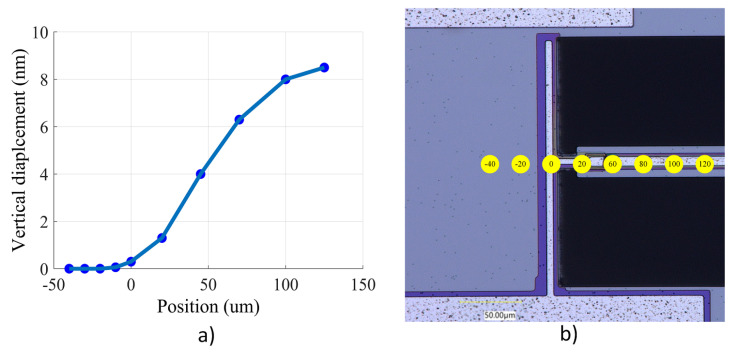
(**a**) Vertical displacement amplitude across the resonator left half measured at the resonating frequency; and (**b**) the SEM picture of half of the clamped–clamped beam resonator with the measurement points where the focus of the vibrometer laser is outlined.

First, to validate the rigidity of the anchors and demonstrate that the anchoring structure does not experience any out-of-plane motion and that the vertical movement on the anchors is negligible, the out-of-plane displacement of the anchoring structure of the device with no holes on its anchoring structure is measured. Figure 8a illustrates the measured vertical displacement of points along the anchoring structure and the beam shown in Figure 8b. In Figure 8a, point zero on the x axis denotes the anchoring point, where the tether connects the resonator to the anchoring structure. It can be observed that there is no vertical movement across the anchoring structure and that the movement amplitude increases toward the center of the beam, as expected by the first mode of resonance of the beam that is being excited. Hence, it is possible to conclude that the 2D model used in this study is appropriate.

### 4.3. Frequency Response

**Figure 9:**
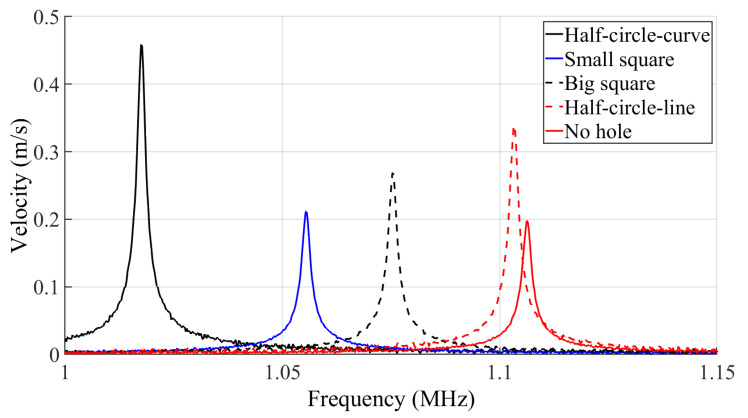
Frequency response of the velocity of the central point of the resonators in air.

**Figure 10:**
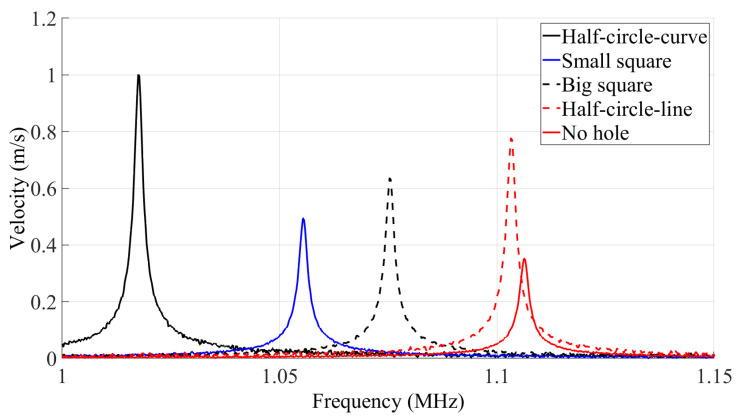
Frequency response of the velocity of the central point of the resonators in vacuum.

Figure 9 and Figure 10, respectively, illustrate the frequency response of the resonator velocity in air and vacuum. The results are shown for each of the five different MEMS beam resonators, denoted according to their reflector geometry type as half-circle-curve, half-circle-line, small square, big square and no hole.

The *Q* is derived from the frequency response of a resonator in both air and vacuum using the ratio of the resonant frequency *f* to the bandwidth Δ*f* at 70.7% of the maximum displacement of the frequency response curve.

The results of the measured *Q* in both air and vacuum are listed in Table 4, along with the simulation results from the PML *Q* analysis and the resonant frequency modal analysis. In order to ensure the validity and accuracy of the test results, and minimize the impact of fabrication process variations, the testing procedure for each standalone resonator was repeated on a total of five distinct dies. Subsequently, statistical analysis was performed to calculate the average and standard deviation of the measured *Q*s and resonant frequencies. The relatively small deviations listed in Table 4 serve as evidence of the relatively good repeatability of our measurements, with minimal impact from the fabrication process variation. These small deviations also ensure that the conclusions drawn are valid.

The results show that the incorporation of reflectors on the anchoring structure of the flexural clamped–clamped beam resonators leads to an increase in the *Q*. It is seen that the device with half-circle-curve shape holes yields the highest *Q*. This behavior was foreseen in Figure 5, as the total energy loss of the half-circle-curve device is the lowest.

The frequency response of the resonators shows that the resonance peaks of the resonators with different anchor types vary between 1.024 MHz and 1.108 MHz in vacuum and between 1.021 MHz and 1.101 MHz in air. It can be further seen that each resonator has a unique resonance frequency and amplitude of displacement. As shown in Table 4, the simulated and measured resonant frequency results agree well with each other. The maximum discrepancy observed between the simulations and measurements is 5.5%. Additionally, adding the holes to the anchoring structure has led to a 7.5% reduction in the resonant frequency based on the simulations and a similar 7.8% reduction based on the measured results. These frequency shifts are partly due to the anchoring structure type, as seen in the previously presented simulations, and can also be caused by slight geometrical variations between the beams tested due to fabrication variability.

In order to provide a more representative comparison of the performance of the resonators that have reflective holes on their anchors with the performance the reference resonator that has no holes on its anchors, the *Q* ratio is defined as:

(3) $$QR=\frac{Q_{device}}{Q_{Noholes}}$$

The QR is reported for the results of the *Q* in both air and vacuum in Table 4, denoted as $QR_{air}$ and $QR_{vacuum}$, respectively.

The results clearly indicate that the incorporation of reflectors can play a crucial role in enhancing the (Q) of resonators, and that the hole geometry can have a significant impact. For instance, in Table 4, the geometry of the holes yielded a QR going from 1.33 to 1.72 in vacuum when comparing the small square holes to the better performing half-circle-curve holes. This is a significant impact as the percentage increase in (Q) is more than doubled when using half-circle-curve holes instead of small-square holes.

It is worthwhile to note that both the PML and measurements support the results of the 2D low-reflecting boundary method, indicating that the method is effective when the elastic wave traveling toward the thickness of the resonator is negligible. Additionally, according to the ideal beam theory, the movement and slope of displacement are assumed to be zero at the endpoints of the resonators. This assumption is supported by both the measurements and the FEM model used in this study.

Table 4 summarizes the results of this study using the FEM method. While the PML model predicts variations in the *Q*, it does not provide insights into the cause of these changes. Since determining the cause of changes in the *Q* is crucial in this study, a 2D wave propagation model was implemented. This model revealed that the presence of trenched holes in the vicinity of the anchoring points leads to the reflection of energy back towards the resonator with different degrees of effectiveness, depending on the hole geometry. In order to check the effectiveness of the results of the method in this work we compared our results with the results of the published works that used the PnC method.

## 5. Conclusions

In this work, the impact of the anchoring structures on the wave propagation in the MEMS clamped–clamped beam flexural mode resonators was investigated with the goal of reducing the anchor loss and in turn increasing the *Q*. Specifically, the effect on the anchor loss of reflectors made of arrays of holes and placed near the anchors was investigated. In this context, a group of clamped–clamped beam resonators were implemented. Different hole shapes were implemented in the arrays of holes comprised within the reflectors, and the results were compared with a reference resonator without reflectors.

The PML method was utilized to investigate the *Q* of the resonators. This indicated that the *Q* for resonators with reflectors was improved with respect to the reference device, with the degree of improvement depending on the hole shape of the reflectors. Notably, this study showed that the half-circle-curve shaped holes led to the highest improvement in the *Q*.

Furthermore, to achieve a deeper understanding of the mechanism behind this enhancement, a 2D numerical model was proposed. In this model, the anchor edges were considered to be low-reflective boundaries and the sum of the energy loss was calculated at these boundaries. This allowed the study of the effectiveness of the different hole geometries in preventing the waves from dissipating to the substrate. The model indicated that the half-circle-curve holes caused the lowest energy escape from the anchoring structure, in agreement with the PML analysis.

The PiezoMUMPS process was used to fabricate the resonators and they were characterized. The measurements corroborated the simulations, with a significant improvement in *Q* observed in the resonators with reflectors. The highest QR was attained using the half-circle-curve hole geometry, showing an improvement in the *Q* by 1.7-fold in the air and 1.72-fold in vacuum, and corroborating the trend seen in the simulation models.

The proposed wave propagation model and the findings of this study have the potential to impact the design and optimization of resonators in a wide range of fields and applications pertaining to MEMS resonators. Importantly, this work shed light on the impact of the reflector hole geometry on the *Q* and provided a 2D numerical model in addition to the more conventional PML method. Future work will focus on optimizing the dimensions of the reflective holes to further increase the performance of the resonators, and on investigating the application of the method to other resonator geometries.

## Figures and Tables

**Table 1:** Dimensions of the resonator and reflector holes.

Feature	Size (μm)
Resonator length (L_R_)	200
Resonator width (W_R_)	25
Tether length	12.5
Tether width	10
Piezoelectric layer width	15
Metal electrode width	7
Big-square side	8
Small-square side	6
Half-circle diameter	8
Arrays pitch (both directions)	11

**Table 2:** The visualization of the wave propagation from the anchor to the substrate at three different times.

	t = 0.5 μs	t = 3 μs	t = 5 μs
No hole	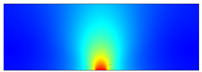	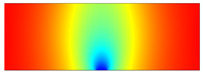	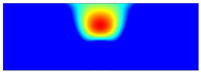
Small square	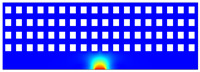	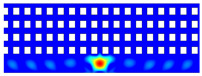	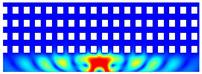
Big square	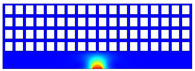	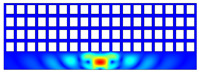	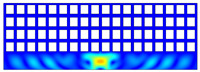
Half-circle-curve	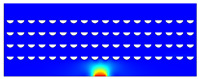	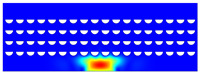	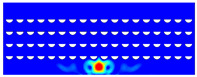
Half-circle-line	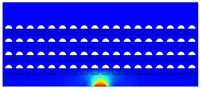	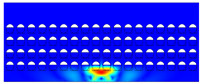	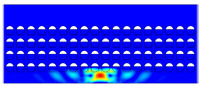

Note: The anchored edge of the beam is at the bottom of each image.

**Table 3:** Impact of the hole geometry on the *Q*, as obtained by PML analysis.

Reflector Holes Shape	($Q_{PML}$)
Half-circle-curve	4131
Half-circle-line	3888
Big square	3623
Small square	3361
No holes	2659

**Table 4:** Impact of the reflector hole geometry on the *Q* of the resonators.

	Hole Type	Half-Circle-Curve	Half-Circle-Line	Big Square	Small Square	No Holes
Simulation	Res. freq. (MHz)	1.029	1.102	1.091	1.064	1.103
	*Q*_PML_	4131	3888	3623	3361	2659
Measurements in air	Res. freq. (MHz)	1.021 ± 0.0022	1.042 ± 0.0015	1.078 ± 0.0027	1.085 ± 0.0021	1.101 ± 0.0023
	*Q*_air_	1620 ± 24	1480 ± 21	1340 ± 24	1240 ± 19	940 ± 21
	QR _air_	1.70	1.57	1.42	1.31	1
Measurements in vacuum	Res. freq. (MHz)	1.024 ± 0.0022	1.048 ± 0.0015	1.082 ± 0.0023	1.092 ± 0.0018	1.108 ± 0.0025
	*Q*_vacuum_	3630 ± 31	3450 ± 44	3100 ± 51	2800 ± 39	2100 ± 46
	QR _vacuum_	1.72	1.64	1.47	1.33	1

## Data Availability

Data underlying the results presented in this paper are not publicly available at this time but may be obtained from the authors upon reasonable request.
